# Identifying the dynamics of actin and tubulin polymerization in iPSCs and in iPSC-derived neurons

**DOI:** 10.18632/oncotarget.22571

**Published:** 2017-11-15

**Authors:** Valentina Magliocca, Stefania Petrini, Tiziana Franchin, Rossella Borghi, Alessia Niceforo, Zeinab Abbaszadeh, Enrico Bertini, Claudia Compagnucci

**Affiliations:** ^1^ Department of Neuroscience, Unit of Neuromuscular and Neurodegenerative Diseases, Laboratory of Molecular Medicine, Bambino Gesủ Children’s Research Hospital, IRCCS, Rome 00146, Italy; ^2^ Confocal Microscopy Core Facility, Research Laboratories, Bambino Gesủ Children’s Research Hospital, IRCCS, Rome 00146, Italy; ^3^ Research Laboratories, Bambino Gesủ Children’s Research Hospital, IRCCS, Rome 00146, Italy; ^4^ Department of Science-LIME, University “Roma Tre”, Rome 00146, Italy

**Keywords:** actin filaments, microtubules, 3D image analysis, iPSCs, neuron

## Abstract

The development of the nervous system requires cytoskeleton-mediated processes coordinating self-renewal, migration, and differentiation of neurons. It is not surprising that many neurodevelopmental problems and neurodegenerative disorders are caused by deficiencies in cytoskeleton-related genes. For this reason, we focus on the cytoskeletal dynamics in proliferating iPSCs and in iPSC-derived neurons to better characterize the underpinnings of cytoskeletal organization looking at actin and tubulin repolymerization studies using the cell permeable probes SiR-Actin and SiR-Tubulin. During neurogenesis, each neuron extends an axon in a complex and changing environment to reach its final target. The dynamic behavior of the growth cone and its capacity to respond to multiple spatial information allows it to find its correct target. We decided to characterize various parameters of the actin filaments and microtubules. Our results suggest that a rapid re-organization of the cytoskeleton occurs 45 minutes after treatments with de-polymerizing agents in iPSCs and 60 minutes in iPSC-derived neurons in both actin filaments and microtubules. The quantitative data confirm that the actin filaments have a primary role in the re-organization of the cytoskeleton soon after de-polymerization, while microtubules have a major function following cytoskeletal stabilization. In conclusion, we investigate the possibility that de-polymerization of the actin filaments may have an impact on microtubules organization and that de-polymerization of the microtubules may affect the stability of the actin filaments. Our results suggest that a reciprocal influence of the actin filaments occurs over the microtubules and *vice versa* in both in iPSCs and iPSC-derived neurons.

## INTRODUCTION

Genetic investigation in humans have revealed that several mutations occurring in genes involved in the development of a functional cytoskeleton are fundamental for the correct neurologic development of the nervous system. The cytoskeleton is involved in a broad series of events regulating neurogenesis and maintenance of the neuronal function, therefore, alterations of genes controlling cytoskeletal dynamics lead to severe neurological diseases. A crucial step in the assembly of motor circuits and in the execution of coordinated movements relies on the accurate navigation of developing axons towards their correct neuronal or muscle targets. Imprecise or ectopic connections of motor axons during development can lead to locomotor or movement disorders (e.g., Congenital Mirror Movements, Hereditary Spastic Paraplegia) [1, 2]. For example, mutations in the genes DCX (DOUBLECORTIN) and LIS1 (LISSENCEPHALY 1), encoding for microtubule associated proteins, are associated with migration defects leading to type I lissencephaly, which consists in the lack of development of brain folds (gyri) and grooves (sulci) [3, 4]. In addition to defects in the development of the nervous system, alterations of the cytoskeleton can affect the maintenance of a functional neuronal network, and therefore leading to neurodegenerative disorders. In fact, several evidences indicate that tubulin acetylation is involved in neurodegenerative diseases, such as Huntington’s disease (HD) and Parkinson’s disease (PD) [5-7]. Moreover, the disarray of microtubules and actin filaments represents one of the early events in the degenerative process of neurons exposed to oxidative stress [8-11]. Importantly, the exact sequence of events leading to neuronal death as well as the molecular determinants for the “dying back” type of axonopathy (where progressive axonal degeneration begins distally and spreads proximally to the cell body), is still obscure and no therapy currently exists to treat the neurodegenerative progression.

The cytoskeleton of eukaryotes is composed of filamentous proteins belonging to three families: 1) the microtubules or MTs (25nm of diameter, made by dimers of α and β-tubulin), 2) the actin filaments or AF (6nm of diameter) and 3) the intermediate filaments or IF (10nm of diameter) [12]. While actin was for long considered as the only cytoskeleton component involved in growth cone steering, recent studies have demonstrated that also MTs are relevant in this process [13-15].

Actin filaments are composed of actin monomers [16], and MTs are polymers composed of alpha- and beta-tubulin heterodimers that stochastically switch between polymerization and de-polymerization, a process known as dynamic instability [17, 18]. Due to their peripheral location within the growth cone and their role in cell migration [19], actin filaments were attributed the leading role in growth cone steering, while MT remodeling was assumed to occur secondarily. Recently, several studies demonstrated that the regulation of MT dynamics on one side of the growth cone is sufficient to induce growth cone turning and, that MTs are, like actin filaments, direct targets of guidance cues in the control of axon navigation [15, 20-23]. For these reasons, our work focuses mainly on actin filaments and on MTs. Their formation, stability and destruction are carefully regulated and are responsible for the high cytoskeletal dynamism that characterize a proliferating, differentiating and differentiated cells. Actin monomers can be added to either end, but changes in the equilibria of polymerization dynamics depend on whether ATP or ADP is associated with actin. MTs are polarized structures that are composed of α- and β-tubulin dimers and are assembled into linear arrays. GTP-tubulin dimers are added to the plus end, and GDP-tubulin dimers dissociate from the minus end following GTP hydrolysis. In growth cones, MT plus ends, which face outwards towards the periphery, exhibit dynamic instability: they cycle through periods of growth, shrinkage and occasional pausing. Growth cone pathfinding is a dynamic process in which the growth cone progresses, pauses, turns and retracts as it navigates through the embryonic landscape and encounters various directions for its travel. The growth cone engages its cytoskeleton to drive forward and turn, continuously progressing through dynamic changes [reviewed in 24]. This occurs thanks to the dynamic properties of actin, that is considered the central part of the mechanism that controls growth cone exploration. Thanks to the increased technological advances in live-cell imaging we have documented how actin flow relates to neurite motility and protrusion following de-polymerization in proliferating induced pluripotent stem cells (iPSCs) and in iPSC-derived neurons. Several studies show that filopodia function as points of attachment to the substrate and produce tension that is used for growth cone progression [23, 25]. The role of the MTs during growth cone steering clearly requires the participation of, and interaction with, actin [26, 27]. Recent live-cell imaging studies show that the function of actin dynamics might provide spatio-temporal guidance to MTs to steer the growth cone in the right direction. Importantly, perturbation of actin structures leads to the redistribution of MTs and a change in the axonal direction of growth [28]. The correct pathfinding of axonal growth cones depends on the dynamic reorganization of both actin filaments and MTs in response to guidance cues. Therefore, understanding how changes in F-actin are coordinated with changes in MTs in response to guidance cues is compulsory to properly unveil the mechanisms of cytoskeletal dynamics. Our present study takes advantage of the utility of cell-permeable chemically synthesized probes SiR-Actin and SiR-Tubulin in following the cytoskeletal dynamism and in the determination of quantitative parameters useful to understand the highly coordinated and integrated interactions between actin filaments and MTs in proliferating iPSCs and in iPSC-derived neurons. Moreover, our results suggest that the de-polymerization of the actin filaments has an effect on the integrity of the MTs and *vice versa* in both iPSCs and in iPSC-derived neurons.

## RESULTS AND DISCUSSION

To study the polymerization rate and the dynamic features of the cytoskeletal components in proliferating iPSCs and in iPSC-derived neurons, we induced de-polymerizations of actin and tubulin filaments by treatment with cytochalasin D or nocodazole, respectively. We analyzed the morphological features of actin and tubulin filaments in live cells using the Filament Tracer interface for automatic detection and segmentation of dendrites and spines.

### Actin dynamics in proliferating iPSCs

**Figure 1:**
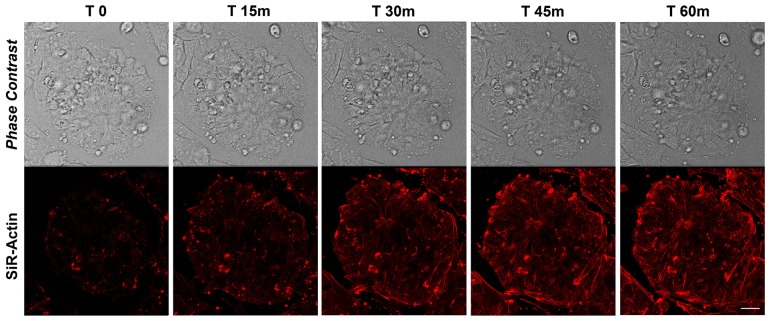
Time-lapse of SiR-Actin probe in proliferating iPSCs Confocal microscopy images with their corresponding bright field photographs of iPSCs following 60 minutes from de-polymerization of the actin filaments with cytochalasin D stained with the live-cell imaging probe SiR-Actin (*red*). Bar: 20 μm.

The actin dynamics in proliferating iPSCs has been analyzed following de-polymerization of the actin filaments with cytochalasin D and the re-polymerization has been monitored with live-cell imaging technology using the cell-permeable Sir-Actin probe. In particular, cytochalasin D is a cell permeable fungal toxin that binds to the barbed end of actin filaments inhibiting both the association and dissociation of subunits. This compound causes the disruption of actin filaments and inhibition of actin polymerization. Silicon-Rhodamine (SiR)-actin is a fluorogenic, cell-permeable and highly specific probe for staining endogenous F-actin. The cells have been monitored for more than 90 minutes following de-polymerization (data not shown), but we decided to report the first 60 minutes as no changes in SiR-Actin distribution nor intensity were observed after this timing (Figure 1). The results obtained show a highly dynamic repolymerization of actin filaments in iPSCs 45 minutes following cytochalasin D treatment (Figure 1)

### MT dynamics in proliferating iPSCs

**Figure 2:**
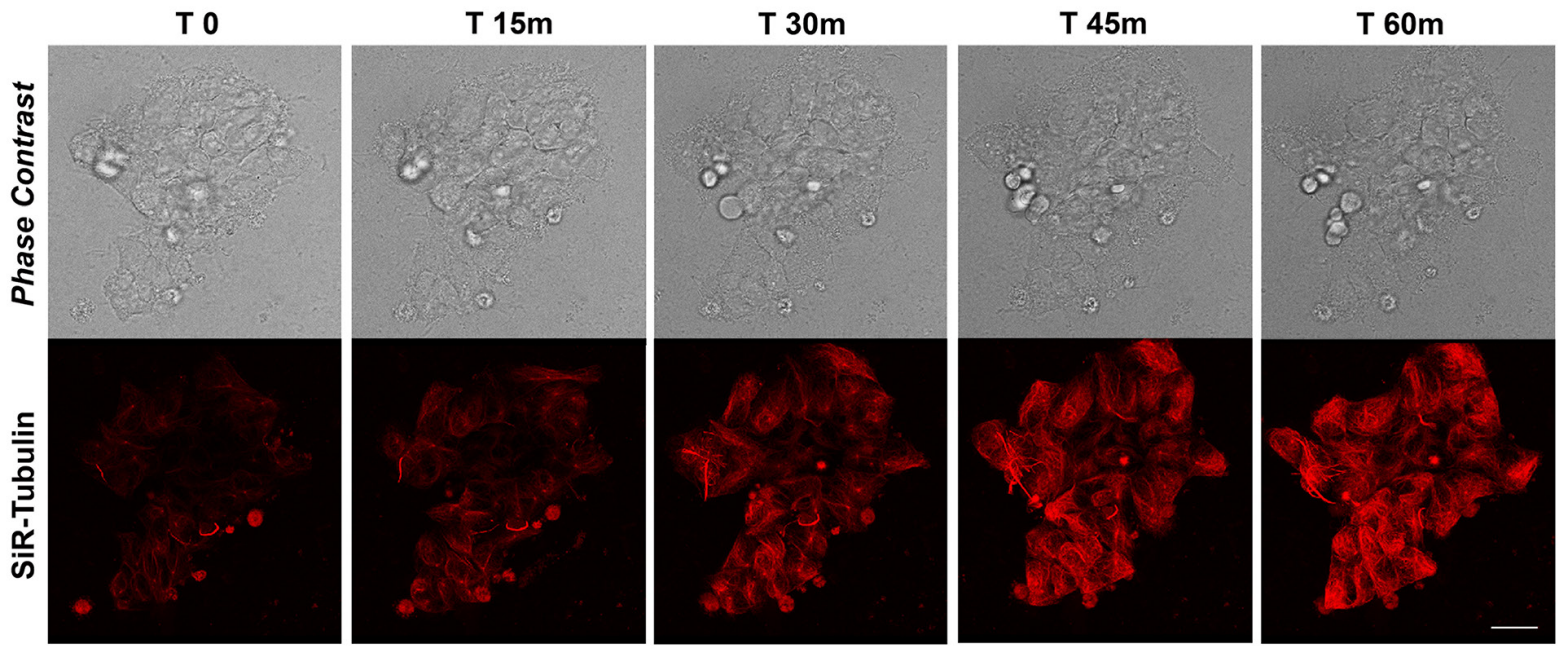
Time-lapse SiR-Tubulin probe in proliferating iPSCs Confocal microscopy images with their corresponding bright field photographs of iPSCs following 60 minutes from de-polymerization of the MTs with Nocodazole stained with the live marker SiR-Tubulin (*red*). Bar: 20 μm.

**Figure 3:**
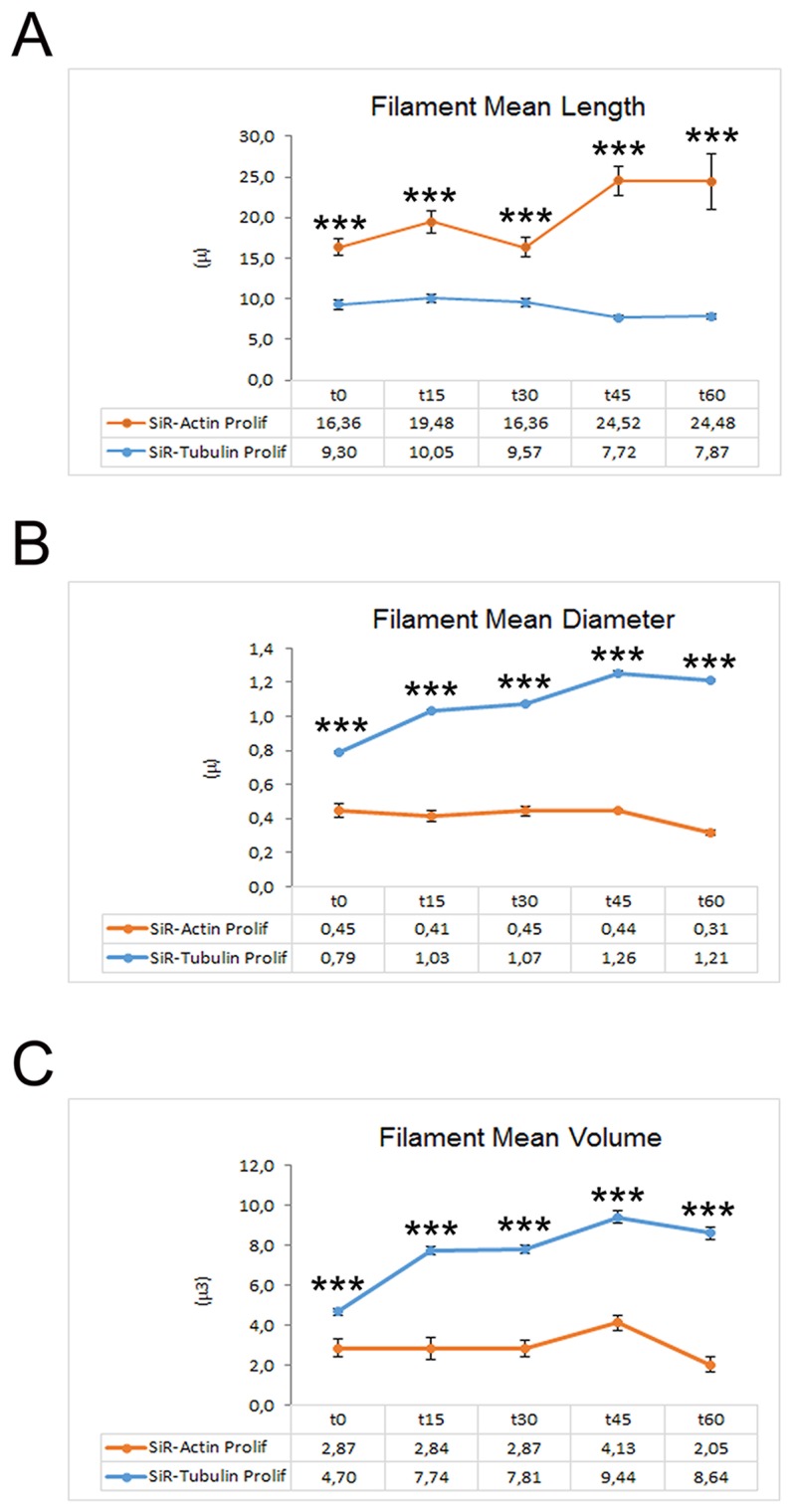
Graphs depicting the quantitative data of actin and tubulin dynamics in proliferating iPSCs obtained by Imaris - Filament analysis module Graphs showing the Filament Mean Length **(A)**, the Filament Mean Diameter **(B)** and the Filament Mean Volume **(C)**. Significant differences were indicated by asterisks (^*^: p< 0,05; ^**^:< 0,01; ^***^: p< 0,001).

The rate of MT polymerization has been investigated following de-polymerization with nocodazole and Sir-Tubulin was used to study the re-polymerization in proliferating iPSCs for more than 90 minutes. Nocodazole is an antimitotic agent that disrupts microtubules by binding to β-tubulin and preventing formation of one of the two interchain disulfide linkages, thus inhibiting microtubule dynamics. SiR-Tubulin live-cell staining is based on the fluorophore silicon rhodamine (SiR) and the microtubule binding drug Docetaxel. As for the actin filaments, the MT dynamics did not change after the first 60 minutes, therefore we decided to report the recoding from time 0 to 60. The time-lapse recording with SiR-tubulin shows that following 60 minutes, the polymerization is completed (Figure 2). As for the actin filaments, the iPSCs microtubules appear to recover their polymerized status 45 minutes following treatment with the depolymerizing agent nocodazole (Figure 2). In particular, the analysis of a single cells instead of the full colony (showed in Figure 2) shows that following de-polymerization, the MT do not reach the cell pseudopodia (Supplementary Figure 1). In line with this last observation are the quantitative data obtained with the Filament module of the Imaris software. We measured the Filaments Mean Length, Filament Mean Diameter and the Filament Mean Volume (Figure 3A-3C) and the data obtained demonstrate that Actin filaments are longer compared to MTs at all time observed (Figure 3A), while the diameter and the volume of the MTs are major than those of the Actin filaments (Figure 3B, 3C).

### Actin dynamics in differentiated iPSC-derived neurons

**Figure 4:**
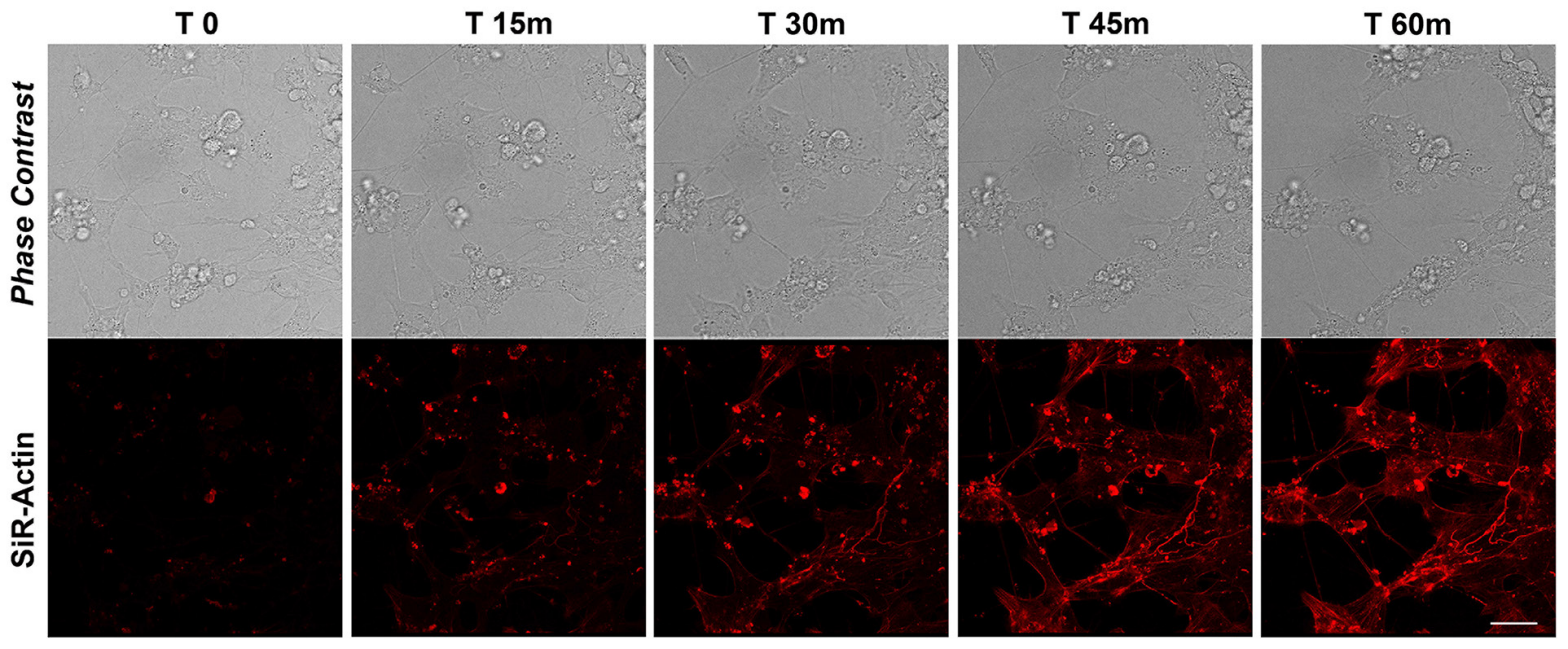
SiR-Actin in differentiated iPSCs Live-cell imaging of SiR-Actin probe in iPSCs-derived neurons. Confocal microscopy images with their corresponding bright field photographs following 60 minutes from de-polymerization of the actin filaments with cytochalasin D stained with the live marker SiR-Actin (*red*). Bar: 30 μm.

**Figure 5:**
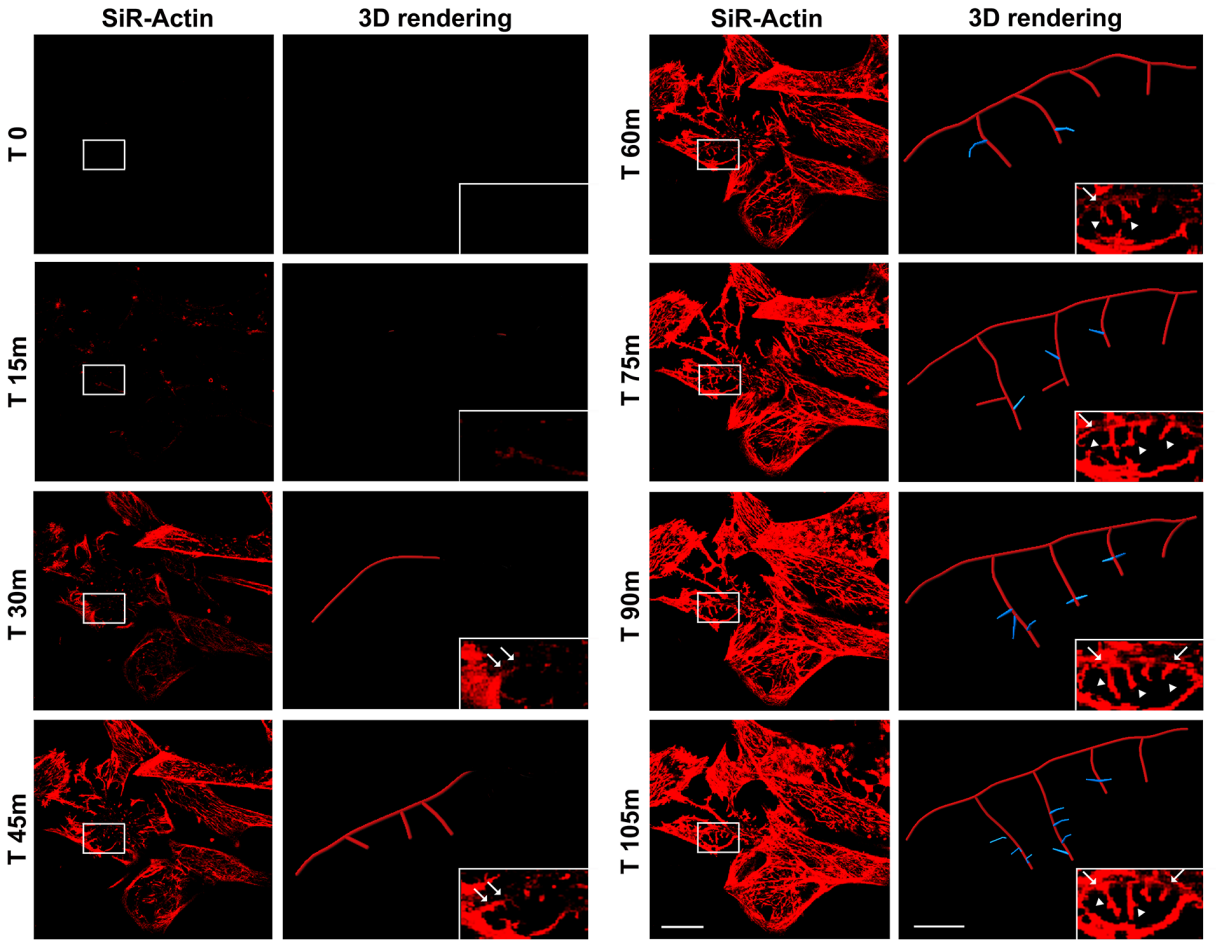
Time-lapse of SiR-Actin probe in iPSC-derived neurons (*left column*) and 3D rendering (*right column*) of dendrites and spines, visualized in high magnification of the insets, using Imaris - Filament module Confocal microscopy images with their corresponding 3D rendering following 105 minutes from de-polymerization of the actin filaments with cytochalasin D stained with the live-cell marker SiR-Actin (*red*). Bars: 20 μm (left) and 2,5 μm (right) respectively.

To monitor the actin dynamics following neural differentiation (Supplementary Figure 2), we tracked the re-polymerization of the actin filaments after cytochalasin D treatment using SiR-Actin live imaging probe. As for the proliferating iPSCs, the cells have been recorded for more than 90 minutes after de-polymerization, but already at 60 minutes no substantial changes were observed, therefore we report images for the first 60 minutes and quantitative data for the first 75 minutes (Figures 4 and 7). The data obtained show that active actin repolymerization occurs between 45 and 60 minutes following repolymerization in iPSC-derived neurons (Figure 4). As our intent is to characterize the changes in the actin dynamics of differentiated neurons comparing the cytoskeletal rearrangements due to actin reorganization versus those to MTs, we reconstructed the morphology of dendritic spine in 3D using the Imaris - Filament module software. In fact, there are no studies regarding the detailed structure of human dendritic morphology (including the distribution and type of spines) obtained *in vitro* from human iPSCs. Electron microscopy can be used to measure dendritic spine structure, but it is time consuming and difficult, making it challenging to obtain large number of measurements. Light microscopy technique is instead limited by the lower level of contrast and resolution of details, but it remains the method of choice to obtain large-scale spatial information regarding the number and distribution of dendritic spine along the dendrites (See Supplementary Video 1). Figure 5 shows examples of 3D reconstruction after apical and basal Z-stacks of dendrites stained with Sir-Actin during re-polymerization experiments and for each stack/time, several morphological parameters were measured following Imaris analysis.

### Tubulin dynamics in differentiating iPSC-derived neurons

**Figure 6:**
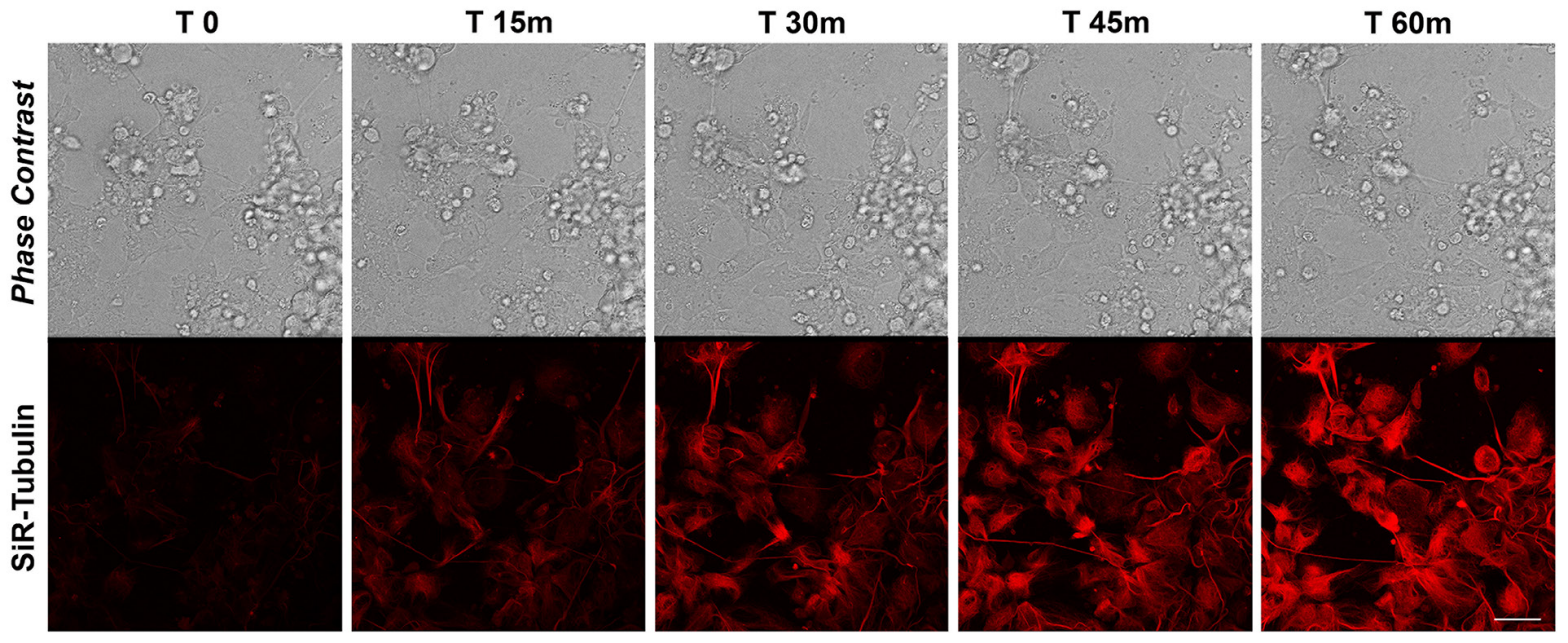
SiR-Tubulin in differentiated iPSCs Time-lapse SiR-Tubulin probe in iPSCs-derived neurons. Confocal microscopy images with their corresponding bright field photographs following 60 minutes from de-polymerization of the MTs with Nocodazole stained with the cell-permeant marker SiR-Tubulin (*red*). Bar: 30 μm.

**Figure 7:**
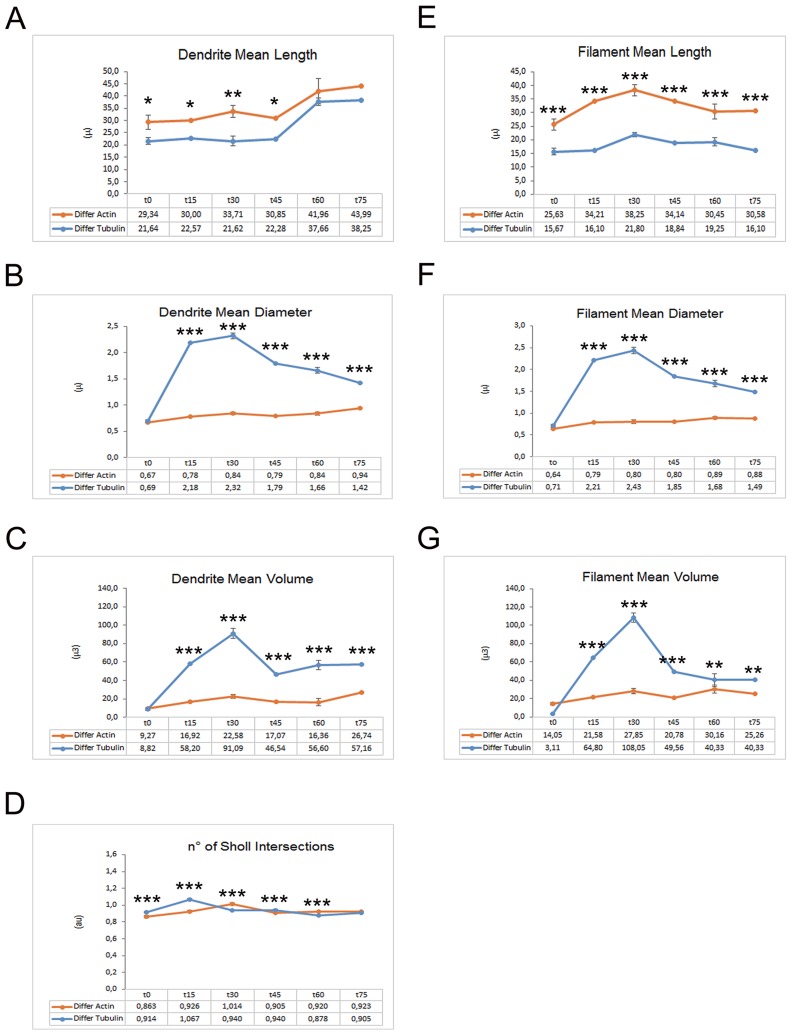
Graphs reporting quantitative data of actin and tubulin dynamics in iPSC-derived neurons obtained by Imaris - Filament analysis module Graphs showing the Dendrite Mean Length **(A)**, the Dendrite Mean Diameter **(B)**, the Dendrite Mean Volume **(C)**, the no. of Sholl Intersections **(D)**, the Filament Mean Length **(E)**, the Filament Mean Diameter **(F)** and the Filament Mean Volume **(G)**. Significant differences were indicated by asterisks (^*^: p< 0,05; ^**^:< 0,01; ^***^: p< 0,001).

To investigate the tubulin dynamics in iPSC-derived neurons, we performed SiR-Tubulin live-cell imaging following nocodazole treatment for more than 90 minutes. As observed with Sir-Actin recordings, the iPSC-derived microtubules appear to recover their polymerized status 45 minutes following treatment with the depolymerizing agent nocodazole, when long SiR-Tubulin-positive neurites were observed (Figure 6). The quantitative analyses obtained with the Imaris-Filament module software show significative changes in dendritic and spine parameters between Actin filaments and MTs. In particular, we measured the Dendrite Mean Length, the Dendrite Mean Diameter, the Dendrite Mean Volume, the no. of Sholl Intersection, the Filament Mean Length, the Filament Mean Diameter, the Filament Mean Volume (Figure 7A-7D). The data obtained show that both the dendrite and the filament length is major in Actin filaments than in MTs (Figure 7A, 7E), therefore confirming that the actin network and bundles are responsible for the expansion of the growth cone, while the MTs follow the actin elongation axis to become stabilized MTs once the road toward the attractant has been paved or the definitive target has been reached (Figure 11). In contrast with the above-mentioned parameters, the dendrite and filament mean Diameter and Volume are increased in MTs when compared with actin filaments and, interestingly, the MTs appear to have a great increase in diameter and volume in both dendrites and filaments at 15 and 30 minutes following the de-polymerization agent (Figure 7B, 7C, 7F, 7G). This result may suggest that during the reorganization of the cytoskeletal network due to the tubulin components, the MTs necessitate of an increased diameter and volume that once stabilized can be reduced. The no. of Sholl intersection is a parameter indicating the neuronal morphology, and in particular the branching index of neurite ramification, therefore a high number indicates a high ramification level. Our analysis of the Sholl Intersection reveals that despite the numbers obtained with the actin filaments and MTs are similar, they are statistically significant (Figure 7D).

**Figure 8:**
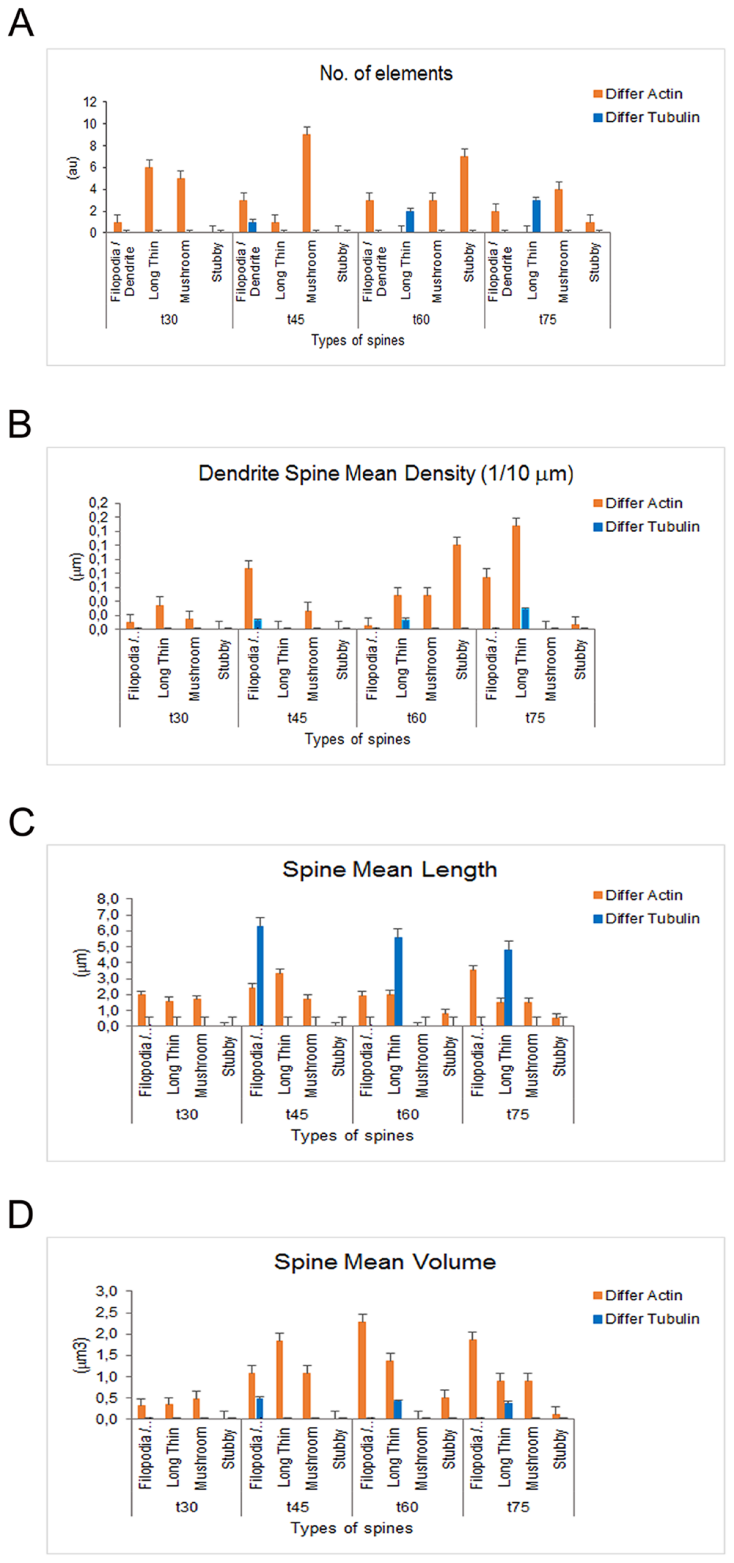
Dendritic spine measurements in iPSCs-derived neurons following actin and tubulin dynamics obtained by Imaris - Filament analysis module Graphs showing the no. of elements **(A)**, the no. of elements (A), the Dendritic Spine Density **(B)**, the Spine Mean Length **(C)** and the Spine Mean Volume **(D)** in iPSC-derived neurons.

**Figure 9:**
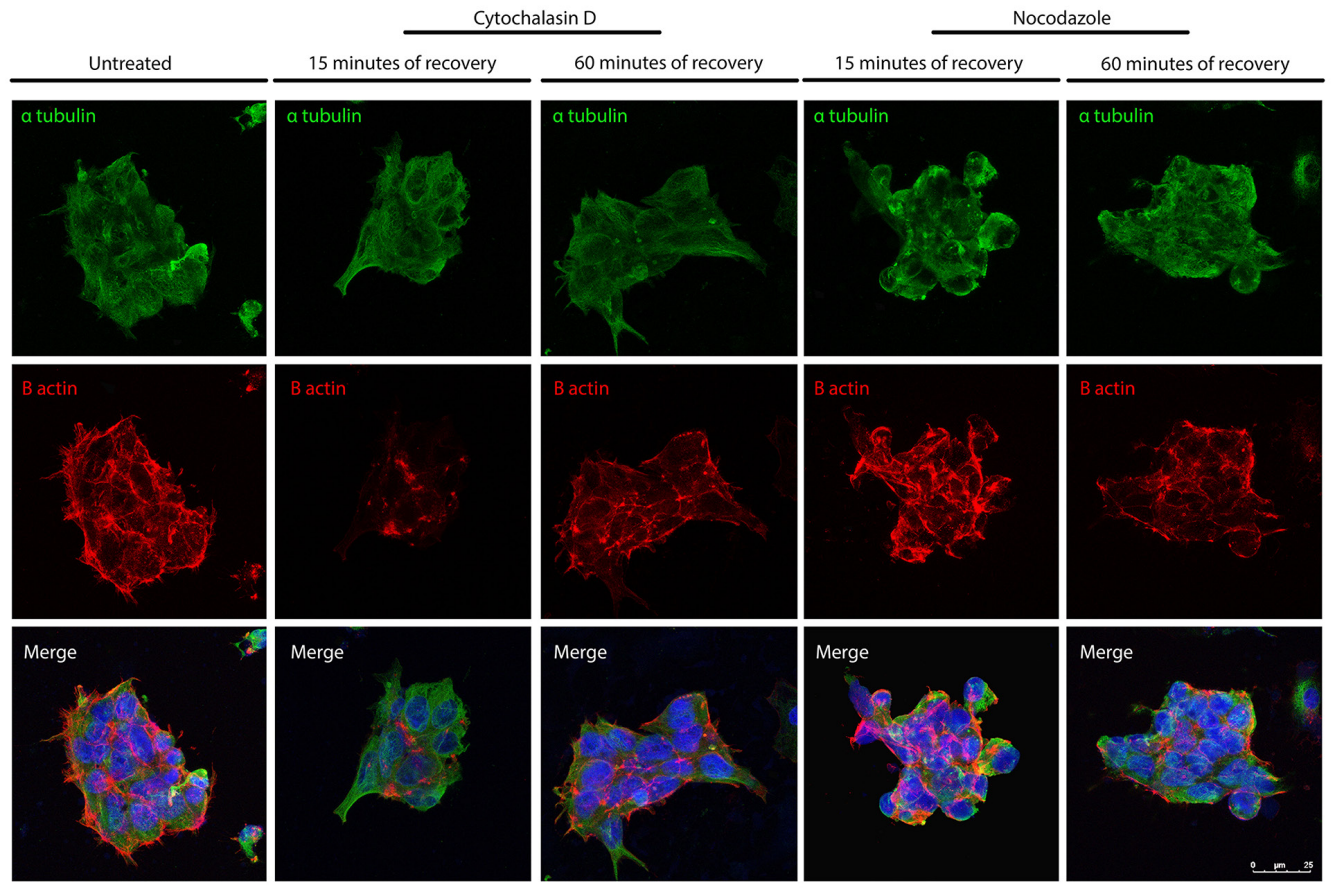
Confocal microscope images showing re-polymerization of microtubules (α-tubulin in *green*) and actin filaments (B-actin in *red*) after cythochalasin D or Nocodazole treatment in iPSCs Bar: 25 μm.

**Figure 10:**
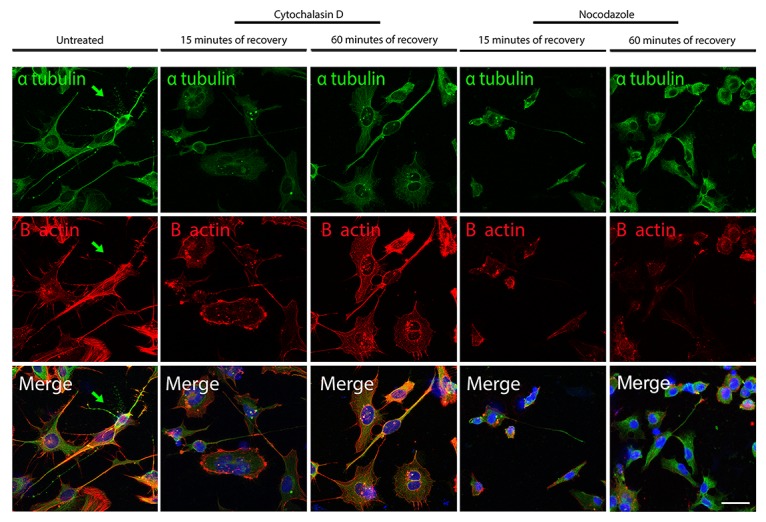
Confocal microscope images showing re-polymerization of microtubules (α-tubulin in *green*) and actin filaments (B-actin in *red*) after cythochalasin D or Nocodazole treatment in iPSCs-derived neurons The arrow point to a neuron with a high α-tubulin signal (*green*) and a weaker B-actin signal, a characteristic common in fully differentiated neurons. Bar: 25 μm.

**Figure 11:**
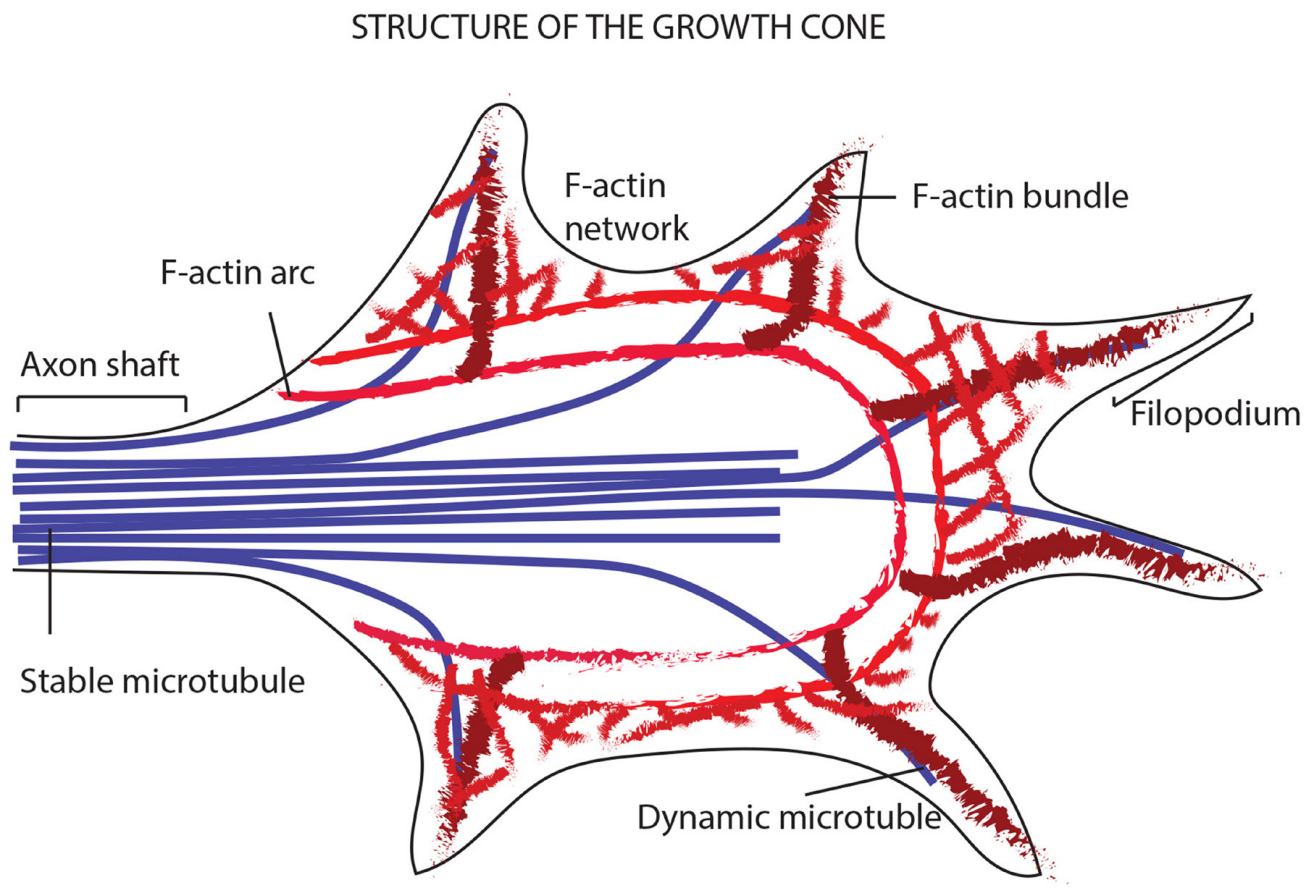
Model showing the way a growth cone senses the neighboring environment and organizes its cytoskeleton in terms of actin filaments and MTs The image shows the leading edge of the growth cone, where filopodia explore the environment thanks to the bundled actin filaments and the F-actin networks (giving structure to the lamellipodia). In addition to the actin filaments, the periphery of the growth cone is made of dynamic MTs that explore the region along F-actin bundles. The core of the growth cone instead, encloses stable bundles MTs that enter the growth cone from the axon shaft. At the zone between the core and the filopodia region, is placed the actomyosin contractile structure (forming the actin arcs).

Regarding the analysis of the spines, the following parameters were measured: No of elements, Dendritic spine Mean Density, Spine Mean Length and Spine Mean Volume (Figure 8). The detailed analyses performed demonstrate that SiR-Actin imaging allows to discriminate between Filopodia/Dendrite, Long thin spines, Mushroom and Stubby spines, while in the SiR-Tubulin recordings only a few type of spines are observed (Figure 8A). The analysis of the Dendrite Spine Mean Density also reveals that actin filaments allow better visualize the spines (Figure 8B), and similar results are obtained with the Spine Mean Volume (Figure 8D). The only parameter where the MT component is prevalent over those of the actin is the Spine Mean Length starting at 45 minutes following nocodazole (Figure 8C). Our interpretation of this result is that once the spine is settled, the tubulin components take over the actin bundles, while during the reorganization of the neuronal cytoskeleton following repolymerization, the actin has a major role in the exploration of the neighboring environment and therefore in the organization of the spines.

### F-Actin equilibrium following MT de-polymerization and MT equilibrium following F-actin de-polymerization in iPSCs

Following the characterization of the actin filaments and MTs re-polymerization and following a detailed quantitative analysis of their parameters, we decided to investigate whether the de-polymerization of the actin filaments has effect on the MTs and if the de-polymerization of the MTs has an impact on the integrity of the actin filaments. Experiments where the iPSCs were treated with cytochalasin D and stained with the α-tubulin and B actin have been performed to investigate the impact that actin filaments de-polymerization has on the MTs (Figure 9). The data obtained demonstrate the efficacy of the de-polymerization agent as B actin signal is very low after 25 minutes of recovery from the treatments. Importantly, the experiment also show that the α-tubulin signal appears diffuse in the cytoplasm after 15 minutes of recovery from the cytochalasin D treatment, a characteristic of disorganized MTs; while following 60 minutes, the localization of the α-tubulin signal is similar to that of untreated cells. We also performed the experiment treating the iPSCs with Nocodazole and staining them with α-tubulin and B actin. The data obtained show that α-tubulin signal is disorganized as expected and, importantly, the B actin signal appears increased if compared with those of the untreated condition. These results suggest that the de-polymerization of the actin filaments has an effect on the integrity of the MTs and *vice versa*.

### F-Actin equilibrium following MT de-polymerization and MT equilibrium following F-actin de-polymerization in iPSC-derived neurons

To investigate the effect of the actin de-polymerization over MTs and of the MTs de-polymerization over the organization of the actin filaments in differentiated iPSCs, we performed the experiments explained above for the iPSCs also on iPSC-derived neurons. The treatment with cytochalasin D lead to a disorganization of the actin filaments demonstrated by the decreased signal of the B actin after 15 minutes of recovery, and importantly a mild delocalization also of the α-tubulin signal. In fact, while the neurites have a strong α-tubulin signal in untreated cells (arrow in Figure 10), after 15 minutes of recovery from the cytochalasin D treatment, the signal in the neurites appears of decreased intensity. Only after 60 minutes of recovery, the α-tubulin is again increased at the level of the neurites. The Nocodazole treatment lead to a decreased α-tubulin signal due to de-polymerization of the MTs and, interestingly, the B actin is also decreased after 15 and 60 minutes of recovery. Therefore, also in iPSC-derived neurons the de-polymerization of the actin filaments has an effect on the integrity of the MTs and the de-polymerization of the MTs has an effect on the actin filament organization.

## MATERIALS AND METHODS

### Cell culture

#### Derivation of human iPSCs (h-iPSCs)

Human fibroblasts obtained from skin biopsies of two healthy patients were purchased from Coriell Institute (USA, Cod GM23338). The iPSCs were derived from human fibroblasts and reprogrammed using the non-integrating episomal technology (Minicircle DNA and mc-iPS Cells, Euroclone, Cat. # SC301A-1).

#### Maintenance and differentiation of iPSCs

Following thawing, iPSCs were grown on MEFs (Life Technologies) for two passages and then in feeder free condition using Matrigel (BD Biosciences) in mTeSR1 (Stemcell Technologies). When the iPSCs were 70–80% confluent, they were passaged 1:4 and transferred to new wells in feeder-free condition and incubated at 37 °C, 5% CO2, the medium was changed every day and the cells split every three days.

#### Differentiation of iPSCs into motor neurons

Motor neuron differentiation has been adapted from (Corti et al., 2012). Cells were plated at a density of 4,2–5,3 × 104 cells/cm2 in Neurocult (StemCell Technologies) for 10 days, then 0,1 μM retinoic acid was added to the cell medium and the medium changed every other day until day 17, when Neurocult was supplemented not only with retinoic acid, but also with 2 μM Dorsomorphin and 3 ng/ml Activin A. On the 24th day the cell medium was replaced with Neurocult supplemented with BDNF (10 ng/ml), GNDF (2 ng/ml), dbcAMP (400 μM), and ascorbic acid (200 μM).

Immunofluorescence analysis of the iPSC-derived neurons was performed as follows: after staining with a cell-permeable probe (i.e. SiR-Actin, see below), samples were fixed with 2% paraformaldehyde in PBS for 10 min, followed by PBS/Triton 0.1% for 5 min., then incubated with an antibody against a neuronal marker, BIII tubulin (1:500, 2h, ^#^5568, Cell Signaling Technology, Danvers, MA). A secondary antibody conjugated with Alexa Fluor-488 (Life technologies, Carlsbad, CA) is used diluted in 1% PBS/BSA for 1h, RT. Nuclei are stained using Hoechst 33342 (Life technologies) according to manufacturer instructions.

#### De-polymerization treatments and SiR-Actin/Sir-Tubulin assays

Cells were plated in μ-Dish ^35mm high^ support and when 70-80% confluent, the cells were treated with 1 μM cytochalasin D (C2618, Sigma Aldrich) for 1 h at 37°C in complete culture medium; after three washes of medium, SiR-Actin probe (SC001, Spirochrome, Cytoskeleton, Denver, CO) is added to the medium at final concentration of 1 μM, then samples are transferred to a laser confocal microscope equipped with a stage incubator for live-cell imaging acquisitions, as described below.

In the MT de-polymerization experiments, Nocodazole has been used as depolymerizing agent (M1404, Sigma Aldrich). Following the treatment with Nocodazole 10 μM for 30 min at 37°C, the cells were washed twice, then stained with 1 μM SiR-Tubulin probe (CY-SC002, Spirochrome).

#### Confocal microscopy and live-cell imaging

Confocal optical sectioning is performed with on a Leica TCS-SP8X laser-scanning confocal microscope (Leica Microsystems, Mannheim, Germany) equipped with a white light laser (WLL) source and a 405nm diode laser. Sequential confocal images are acquired using a HC PLAPO 63x oil-immersion objective (1.40 numerical aperture, Leica Microsystems). Z-reconstructions of serial single optical sections are acquired every 3 min (for SiR-Actin probe) or 5 min (for SiR-Tubulin probe), and carried out with a 512x512 format, scan speed of 400Hz, a pixel size of 0,3 μm, and z-step size of 0,5 μm. Lasers’ power, beam splitters, filter settings, pinhole diameters and scan mode are the same for all examined samples of each staining. Time-lapse microscopy was performed with a stage incubator (OkoLab, Naples, Italy) allowing to maintain stable conditions of temperature, CO_2_ and humidity during live-cell imaging.

Z-reconstructions are imported into Imaris (Bitplane, Zurich, Switzerland) software to obtain their three-dimensional (3D) surface rendering. To improve contrast and resolution of confocal raw images, deconvolution analysis (3D Deconvolution software, Leica Microsystems) is applied to Z stacks before 3D reconstruction. The reconstructed images were assembled in Adobe Photoshop CS6 software (Adobe Systems Inc., San Jose, CA).

#### 3D rendering and quantitative analysis of the morphometrical data

The 3D reconstruction of the filaments from confocal stacks is obtained with Imaris (Bitplane, Zurich, Switzerland) software. Imaris provides the feature Filament Tracer that allows the automatic detection of 3D neuronal filament-like structures, composed by dendrites, axons and spines. Cytoskeleton filaments stained by SiR-Actin or SiR-Tubulin, are individually isolated in space and isosurfaces have been built using the following parameters: individual voxels are defined with a size of 0,3x0,3x0,5μm; a quality threshold (Gaussian filtering) is defined in SiR-Actin or SiR-Tubulin labelled filaments as a pre-processing stage to diminish noise; then raw data are exported in a *Excel* spreadsheet (Microsoft). From 80 to 100 cells were analyzed for condition analyzed and condition analyzed. 3D reconstruction of filament-like structures is performed using automatic detection method with the Autopath option mode that allow to reselect, redraw and create new filament structures. The statistical analysis associated to each filter categories identified by Imaris as filaments, dendrites and dendritic spines, was carried out at different stages of proliferation and differentiation to obtain the following morphological parameters:

1) *length, diameter and volume* measurements of all the filaments segment edges in proliferating iPSCs;
2) *length, diameter and volume* measurements of all segment edges of the dendrites and/or filaments in iPSC-derived neurons;
3) *number of sholl intersections*, defined as number of dendrite intersections (branches) in iPSC-derived neurons;
4) *number, length and volume* of all the spines branching off a dendritic segment in iPSC-derived neurons was obtained through the use of the Filament-Tracer module. *Dendritic spine length* was manually reconstructed starting from its insertion point to the distal tip of the spine;
5) *dendritic spine density*, defined by the number of spines per unit length (10 μm) of dendrite;
6) *shape features of dendritic spines* are measured using Imaris Spine Classifier to quantify spines based on predefined shape parameters as stubby, mushroom, long/thin or filopodia/dendrite shape;
7) *spine head volume* of all spines branching off a dendritic segment in iPSC-derived neurons.

### Statistical analysis

Quantitative results are reported as means ± standard error of the mean (SEM). For comparing overall differences of the extracted 3D filaments morphometric feature data obtained in different samples, the Student’s paired-sample t-test was performed. *P* values less than 0,05 are considered statistically significant, and all reported *p*-values are two sided.

## CONCLUSIONS

The imaging and analysis of distinct cytoskeleton components may have important implications for understanding mechanisms underlying cytoskeletal dynamics in cell proliferation and neuronal differentiation as well as cell polarization and morphogenesis. In this study, we perform time-lapse imaging experiments to record the birth and formation of actin and tubulin cytoskeletal filaments after induced de-polymerization and to directly record spine maturation in living iPSC-derived neurons, comparing differentiation and proliferation status. Using cell-permeant probes specific to detect polymerized actin and α-tubulin, we analyzed nascent F-actin and MTs dynamics specifically, by capturing the *de novo* growth properties of F-actin and MTs over time. After F-actin and MTs de-polymerization with the drug cytochalasin D and nocodazole, respectively, we performed live-cell imaging experiments to record re-polymerization rate of cytoskeleton filaments. Our results show that the quantitative parameters (Filament Mean Length, the Filament Mean Diameter, the Filament Mean Volume, Dendrite Mean Length, the Dendrite Mean Diameter, the Dendrite Mean Volume and the no. of Sholl Intersections) recorded for the re-polymerization of F-actin and MTs are significantly different both during proliferation of iPSCs and in differentiated neurons derived from iPSCs. This confirms that the mechanisms controlling F-actin and MTs re-organization have their own features. Despite these data may suggest that these two cytoskeletal components are independent one to the other, the data obtained with cross experiments where actin distribution was observed following de-polymerization of the MTs and α-tubulin following de-polymerization of the actin filaments, demonstrate that actin filaments and MTs are coordinated and integrated in their distribution and function. In fact, the re-distribution of one lead to an altered localization of the other in both proliferating iPSCs and in iPSC-derived neurons. To complete our studies on the iPSC-derived neurons we analyzed their spines using both cell-permeable SiR-Actin and SiR-Tubulin probes and our results confirm the literature data that actin filaments are responsible for first assessing the neighboring environment [26, 28], and once the spines are settled, the MTs take over the actin bundles (Figure 11). Having used the live 3D reconstruction methodology, we were able to analyze deeply several parameters that otherwise were difficult to obtain in large scale. Therefore, this technique offers great potential in studying cytoskeletal dynamics. For this reason, we envision that several iPSCs models of human disorders would greatly benefit from the application of live 3D reconstruction methodology. In fact, one the problem of using iPSCs is the expensive cost of the growth media, but with this methodology, it is possible to study a great number of parameters at the same time, thus allowing to use the minimal amount of the precious iPSCs material. In conclusion, the present work poses the bases for a careful quantitative analysis performed using for the first time the innovative integration of 3D reconstruction comparing several cytoskeletal components using the *in vitro* model of iPSCs and iPSC-derived neuronal differentiation.

## SUPPLEMENTARY MATERIALS FIGURES AND VIDEO


