# Supplementary material for: Identifying the dynamics of actin and tubulin polymerization in iPSCs and in iPSC-derived neurons
Source: Oncotarget. 2017 Nov 15;8(67):111096–109. doi: 10.18632/oncotarget.22571 (PMC5762308; doi:10.18632/oncotarget.22571)
Supplement: Supplementary file 1 [file oncotarget-08-111096-s001.pdf]

Identifying the dynamics of actin and tubulin polymerization in iPSCs and in iPSC-derived neurons

SUPPLEMENTARY MATERIALS

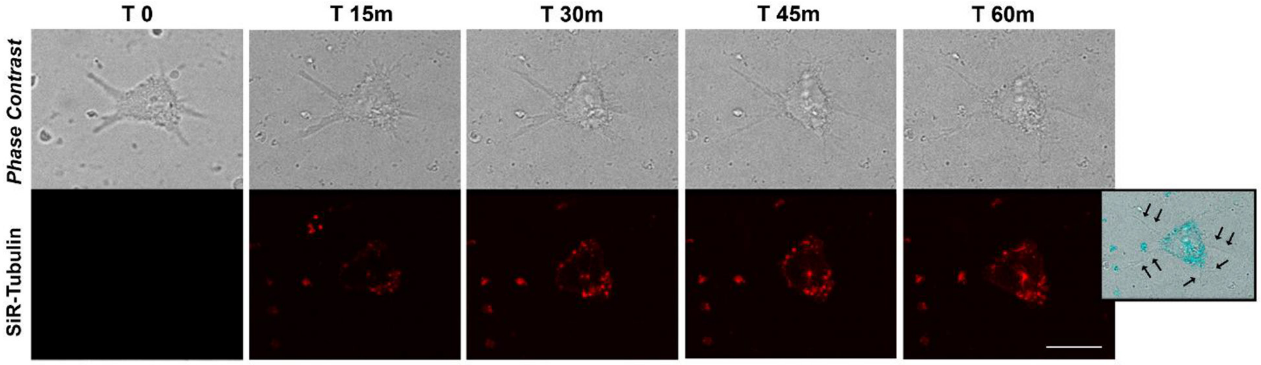

Supplementary Figure 1: A representative proliferating iPSC stained with SiR-Tubulin probe (*red*) exhibiting a slow polymerization rate of tubulin filaments that remain assembled in the cell body and concentrated around the nucleus, and they do not reach the cell pseudopodia (*arrows*), as visualized by the phase contrast image (SiR-Actin pseudo-colored in *cyan*). Bar: 20 um.

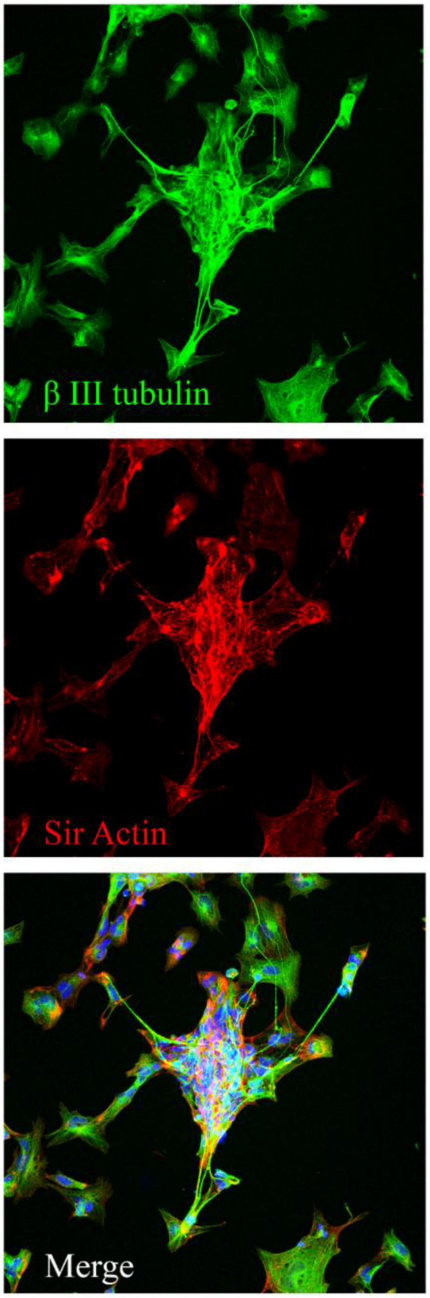

Supplementary Figure 2: Confocal microscope images showing cells positive to the neuronal marker beta III tubulin and SiR-Actin. The figure show that the cells stained for the live-cell imaging probe SiR-Actin are also positive to the neuronal marker, thus demonstrating that the cells analyzed are differentiated.

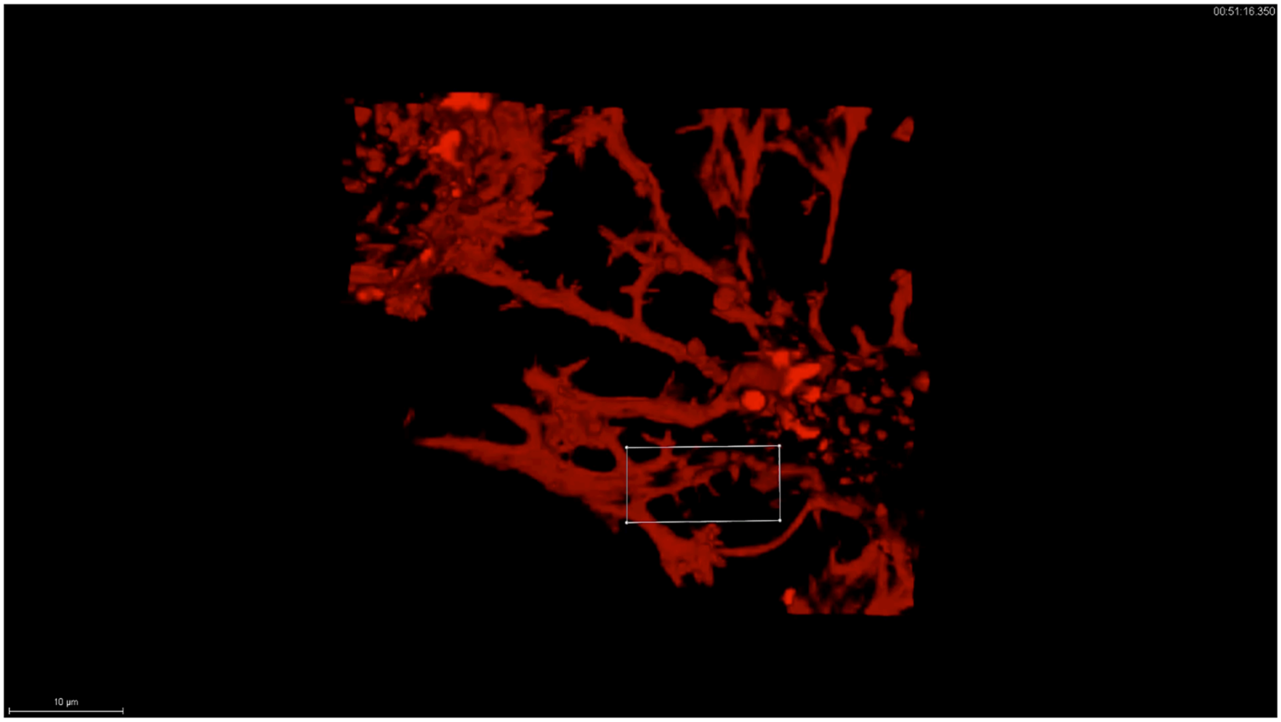

Supplementary Video 1: High magnification of SiR-Actin probe time-lapse in iPSC-derived neurons represented in Figure 5, with the corresponding inset describing the development of dendrites and spines during the acquisition time.
